# A clinical prediction model for complicated appendicitis in children younger than five years of age

**DOI:** 10.1186/s12887-020-02286-4

**Published:** 2020-08-25

**Authors:** Wei Feng, Xu-Feng Zhao, Miao-Miao Li, Hua-Lei Cui

**Affiliations:** 1 Graduate school, Tianjin Medical University, Tianjin, 300070 China; 2 Department of Pediatric Surgery, Tianjin Children’s Hospital, Tianjin, 300134 China

**Keywords:** Acute appendicitis, Complicated appendicitis, Children, Pre-school age

## Abstract

**Background:**

No reliably specific method for complicated appendicitis has been identified in children younger than five years of age. This study aimed to analyze the independent factors for complicated appendicitis in children younger than five years of age, develop and validate a prediction model for the differentiation of simple and complicated appendicitis.

**Methods:**

A retrospective study of 382 children younger than five years of age with acute appendicitis from January 2007 to December 2016 was conducted with assessments of demographic data, clinical symptoms and signs, and pre-operative laboratory results. According to intraoperative findings and postoperative pathological results, acute appendicitis was divided into simple and complicated appendicitis. Univariate and multivariate analyses were used to screen out the independent factors of complicated appendicitis, and develop a prediction model for complicated appendicitis. Then 156 such patients from January 2017 to December 2019 were collected as validation sample to validate the prediction model. Test performance of the prediction model was compared with the ALVARADO score and Pediatric Appendicitis Score (PAS).

**Results:**

Of the 382 patients, 244 (63.9%) had complicated appendicitis. Age, white blood cell count, and duration of symptoms were the independent factors for complicated appendicitis in children younger than five years of age. The final predication model for complicated appendicitis included factors above. In validation sample, the prediction model exhibited a high degree of discrimination (area under the curve [AUC]: 0.830; 95% confidence interval [CI]: 0.762–0.885) corresponding to a optimal cutoff value of 0.62, and outperformed the PAS (AUC: 0.735; 95% CI: 0.658–0.802), ALVARADO score (AUC: 0.733; 95% CI: 0.657–0.801).

**Conclusion:**

Age, white blood cell count, and duration of symptoms could be used to predict complicated appendicitis in children younger than five years of age with acute appendicitis. The prediction model is a novel but promising method that aids in the differentiation of acute simple and complicated appendicitis.

## Background

Acute appendicitis (AA) is the most common surgical disease in children, and its incidence is reported to be increasing [1]. The diagnosis of acute appendicitis has classic clinical appearance only in one third of all patients. Clinical appearance in the in the patients younger than five years of age is often atypical, and misdiagnosis in this age group is not rare, which can lead to an increased rate of perforation [2]. Clinical presentation, ALVARADO score, Pediatric Appendicitis Score (PAS), Computed tomography, ultrasound and blood tests, may be helpful in diagnose of AA, but it is difficult to confirm the type of appendicitis (simple or complicated appendicitis), especially for children younger than five years of age [3–7]. Been able to diagnose simple vs. complicated appendicitis allows the surgeon to choose the best surgical approach ranging from antibiotics and delayed appendectomy to laparotomy [8–10]. Perforated appendicitis after surgery requires antibiotic mono or combination therapy [11]. Determining the optimum algorithm for diagnostic procedure in complicated AA may not only reduce the number of unnecessary operations, but also the frequency of complications, and may contribute significantly to reducing the cost of treating patients with acute abdominal conditions. There are tools to determine the severity of AA (abdominal ultrasound and computed tomography); nevertheless, this tools may be limited in some centers e.g. technicians that can not give a final report or lack of personnel to carry them out [12]. Consequently, simple and efficient methods to estimate the complicated appendicitis are currently of interest.

At present, several effective methods have been reported for predicting complicated appendicitis in children with AA, but it is malfunctioning in patients younger than five years of age [6, 7, 13, 14]. Therefore, it is important to predict the type of AA accurately in children younger than five years of age, in order to choose the optimal treatment strategy and save medical resources. Thus, the present study investigated the clinical and laboratory data to screen out the independent factors of complicated appendicitis, develop and validate a prediction model to differentiate simple from complicated appendicitis in children younger than five years of age with AA.

## Methods

The Institutional Review Board of Tianjin Children’s Hospital approved the collection and use of the clinical information of the patients for research purposes before the investigation was started and waived the requirement for informed consent. (IRB number L202001). Our primary goal was to develop a clinical prediction model for complicated appendicitis in children younger than five years of age. The secondary goal was to validate the prediction model for the differentiation of simple and complicated appendicitis.

### Settings and children

We reviewed the files of AA patients younger than five years of age in the pediatric surgery department of Tianjin children Hospital from January 2007 to December 2016 as the derivation sample to establish a complicated appendicitis prediction model. And such patients from January 2017 to December 2019 were collected as the validation sample for external verification of the prediction model. The cases of a total of 602 patients younger than five years of age were retrieved initially, all of which were confirmed to be AA by intraoperative findings and postoperative pathological results. The patients had not been treated with antibiotics or other anti-inflammatory drugs before admission. Patients with inflammatory diseases (such as pneumonia, cholecystitis) and previous history of abdominal surgery, treated nonoperatively with antibiotics and drainage procedures because of the formation of a well-defined abscess, and those who had acute onset of chronic appendicitis were excluded from the study. Thus, 64 patients were excluded, and 538 subjects were enrolled for the following study.

### Study design

The characteristics of subjects from derivation sample, including (1) demographic data: age, gender, body mass index (BMI); (2) symptoms and signs: duration of symptoms (DS), body temperature, right lower quadrant (RLQ) tenderness and rebound pain, migration of pain to RLQ, abdominal distention, nausea and (or) vomiting, anorexia, constipation, diarrhea; (3) intraoperative observation and postoperative pathological results, were extracted from inpatient medical records. The white blood cell count (WBC), neutrophil count (NEUT), percentage of neutrophils (PN), lymphocyte count (LYMPH), mononuclear cell count (MC), platelet count (PLT), C-reactive protein (CRP) and procalcitonin (PCT) data tested on admission (within 2 h) in venous blood samples were collected. After the establishment of prediction model for complicated appendicitis, clinical data such as the age, DS, and WBC of validation sample were collected. Furthermore, we performed the ALVARADO score and PAS for patients in validation sample [3]. For these symptoms and signs, “unsure,” “don’t know,” and “missing” responses were coded as not having the sign or symptom [6]. DS was defined as the period from the moment the patient first felt ill (any of fever, abdominal pain, abdominal distention, nausea, vomiting, anorexia, constipation and diarrhea) until the time of admission, as reported by the family members of patients.

AA was divided into simple appendicitis and complicated appendicitis according to the following diagnostic code. Simple appendicitis is diagnosed on the basis of (1) intraoperative findings: inflamed appendix without signs of gangrene, perforation, purulent fluid, contained phlegmone or intra-abdominal abscess and (2) histopathological examination confirming the diagnosis of appendicitis without necrosis or perforation. Complicated appendicitis is diagnosed on the basis of (1) intraoperative findings: signs of a gangrenous appendix with or without perforation, intra-abdominal abscess, appendicular contained phlegmone, or purulent free fluid and (2) histopathology confirming the diagnosis based on extensive necrotic tissue in the muscular layer of the appendix or signs of perforation [7, 9, 15]. In case of discrepancies between clinical and pathological findings, the final result refers to the pathologist.

### Statistical analysis

Excel software was used to data entry, Statistical Package for Social Sciences (SPSS) softwares were used for statistical assessments, and drawing ROC curve with MedCalc 15.0 software. The normal distribution of the data was evaluated with the Shapiro-Wilk test. Values without normal distribution were presented as medians and inter-quartile ranges (IQR). Categorical variables were presented as numbers and percentages. Numerical values in the simple appendicitis group and the complicated appendicitis group were compared using the Mann-Whitney U test. Chi-square test was used in comparison of categorical data. Univariable analysis was utilized in order to determine the effects of potential factors on complicated appendicitis. Significant factors were included in the stepwise multivariate Logistic regression model and independent factors were identified. The complicated appendicitis prediction model was established based on independent factors, and the area under the curve (AUC) of ROC was used to quantify the differentiation degree of the prediction model. In statistical analysis, a *P* < 0.05 with 95% confidence interval (95% CI) and 5% margin of error was considered statistically significant.

## Results

### Study population

**Fig. 1:**
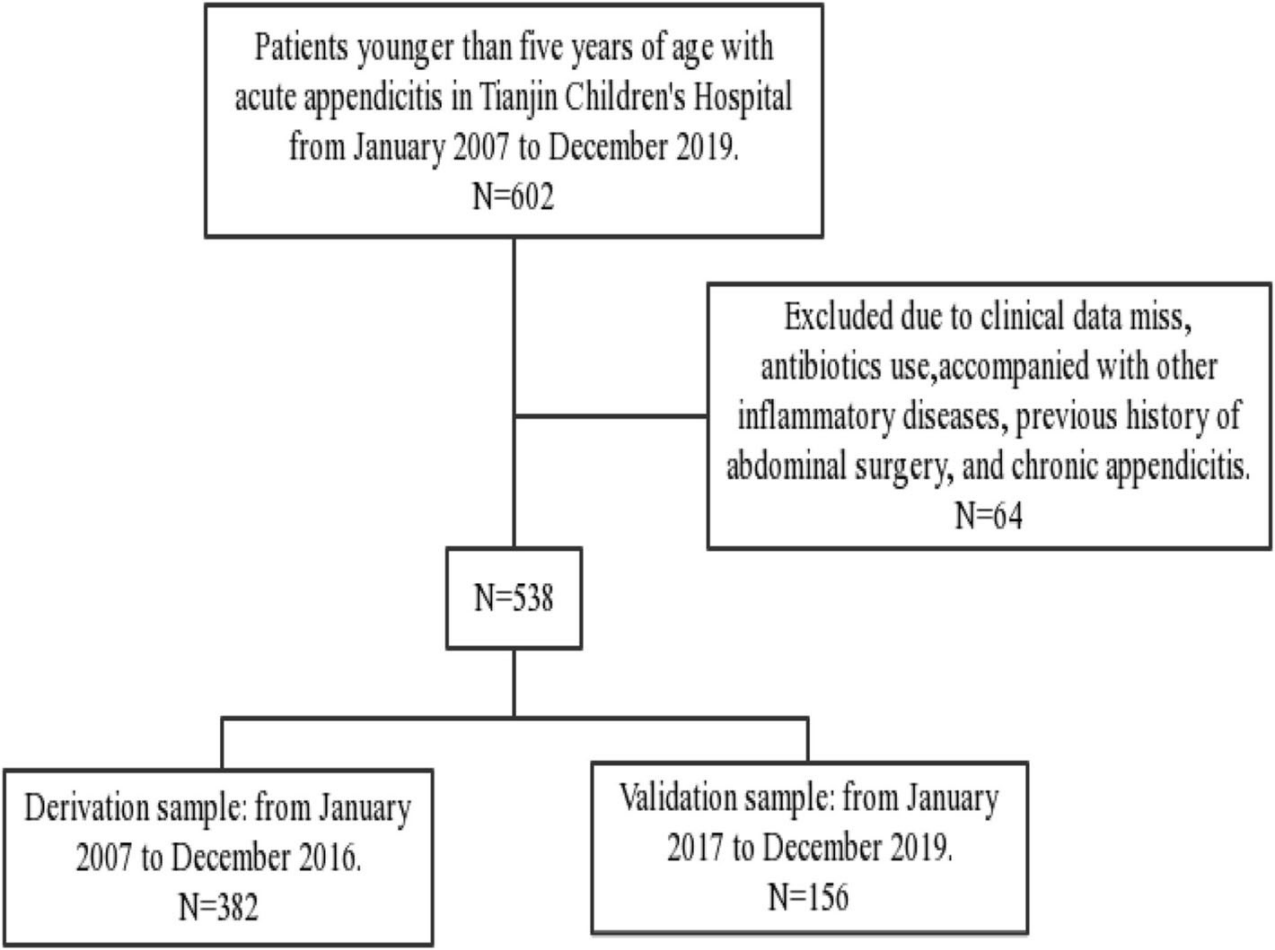
Flow chart of the study population

The entire number of patients met the the inclusion criteria during the time frame of the study was 538. We included 382 patients in derivation sample and 156 patients validation sample (Fig. 1). In derivation sample, there were 224 males (58.6%) and 158 females (41.4%); the age range was 0.1 to 5 years; the duration of symptoms was 4 to 146 h; the body temperature range at admission was 36.6 to 39.3 °C. Among them, 244 cases (63.9%) were complicated appendicitis and 138 cases (36.1%) were simple appendicitis.

### Prediction model development

The demographic data, pre-operative laboratory results, and symptoms and signs of different AA types in derivation sample are listed in Table 1. No significant differences in gender, BMI, PN, MC, PLT, LRQ tenderness, anorexia, or constipation existed between complicated appendicitis and simple appendicitis. Patients with complicated appendicitis were significantly younger, had longer DS, had higher body temperature, and more frequently reported migration of pain to RLQ, abdominal distention, nausea/vomiting, and diarrhea (*P* < 0.05 for all). Comparison of pre-operative laboratory results, median WBC, NEUT, LYMPH, CRP, and PCT level were significantly higher (WBC: 15.8 versus 12.3 [*10^9^/L]; NEUT: 11.8 versus 9.6 [*10^9^/L]; LYMPH: 3.0 versus 2.6 [*10^9^/L]; CRP: 58.5 versus 35.1 [mg/L]; PCT: 0.26 versus 0.12 [μg/L]; *P* < 0.05 for all) in patients with complicated appendicitis than that with the simple appendicitis.

**Table 1:** Univariate analysis of clinical data on the AA types. (Derivation Sample: *n* = 382)

Variables	Complicated appendicitis (*n* = 244)	Simple appendicitis (*n* = 138)	*P* value
**Demographic data**			
Age (years)^#^	3.3(2.5,4.1)	4.4(4.1,4.8)	< 0.001^a^
Male:Female	141:103	83:55	0.667^b^
BMI (kg/m^2^)^#^	23.8(18.3,29.6)	23.7(18.2,29.1)	0.692^a^
**Pre-operative laboratory values**			
WBC (*10^9^/L)^#^	15.8 (13.9,18.7)	12.3 (9.9,15.0)	< 0.001^a^
NEUT (*10^9^/L)^#^	11.8 (9.3,13.5)	9.6 (7.2,12.1)	< 0.001^a^
PN (%)^#^	79.5 (63.2,86.2)	79.0 (72.8,85.1)	0.534^a^
MC (*10^9^/L)^#^	0.88 (0.51,1.21)	0.88 (0.57,1.27)	0.561^a^
LYMPH (*10^9^/L)^#^	3.0 (2.3,5.7)	2.6 (1.9,3.4)	< 0.001^a^
PCT (ug/L)^#^	0.26 (0.08,1.41)	0.12 (0.05,0.42)	< 0.001^a^
CRP (mg/L)^#^	58.5 (20.2124.8)	35.1 (15.9,80.2)	0.002^a^
PLT (*10^9^/L)^#^	279.0 (236.0,331.0)	278.0 (243.5316.8)	0.663^a^
**Clinical findings**			
DS (hours)^#^	38 (24,84)	24 (12,49)	< 0.001^a^
Body temperature (°C)^#^	38.5 (37.6,38.8)	38.1 (37.6,38.7)	< 0.001^a^
Migration of pain to RLQ n (%)	96 (39.3)	16 (11.6)	< 0.001^b^
LRQ tenderness n (%)	196 (80.3)	119 (86.2)	0.163^b^
Abdominal distention n (%)	111 (45.5)	36 (26.1)	< 0.001^b^
Rebound pain n (%)	155 (63.5)	28 (20.3)	< 0.001^b^
Nausea/ vomiting n (%)	139 (57.0)	17 (12.3)	< 0.001^b^
Anorexia n (%)	182 (74.6)	109 (79.0)	0.382^b^
Constipation n (%)	23 (9.4)	22 (15.9)	0.069^b^
Diarrhea n (%)	117 (48.0)	11 (8.0)	< 0.001^b^

^#^Values are presented as medians and inter-quartile ranges; ^a^Mann-Whitney U test; ^b^Chi-square test. *BMI* body mass index, *WBC* white blood cell count, *NEUT* neutrophil count, *PN* percentage of neutrophils, *MC* mononuclear cell count, *LYMPH* lymphocyte count, *PCT* procalcitonin, *CRP* C-reactive protein, *PLT* platelet count, *DS* duration of symptoms, *LRQ* right lower quadrant

**Fig. 2:**
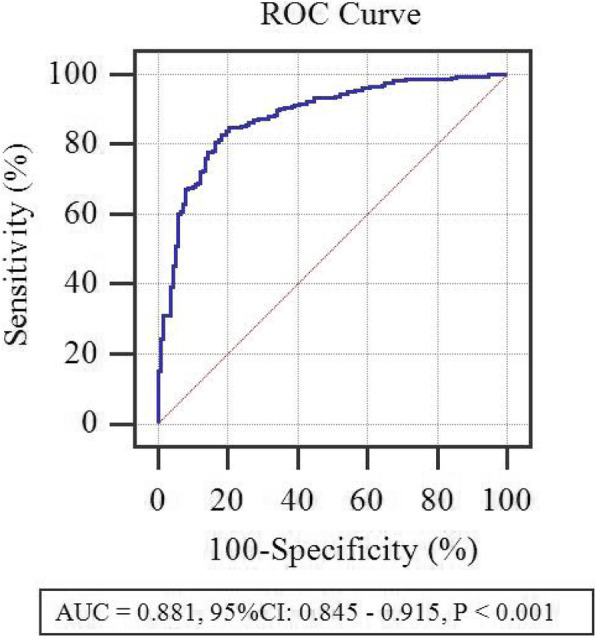
ROC curve of prediction model in derivation sample. The AUC for the prediction model was 0.881 (95% CI: 0.845–0.915)

Significant influenced factors were included in the backward stepwise regression analysis. Age, WBC, and DS were the independent predictors for complicated appendicitis in children younger than five years of age, and these factors were entered into the prediction model (Table 2). Diagnosis of collinearity for the above three variables was performed, and the variance expansion factors were 1.023, 1.076 and 1.072, respectively, suggesting that there was no multiple collinearity relationship. Based on the multivariate regression analysis results, we referred the Enter method (P = Expi∑BiXi/1 + Exp∑BiXi) to establish the regression equation (prediction model): P = e^x^/(1 + e^x^), ‘e’ is the natural logarithm, X = 2.997–1.559 A1 + 0.190 A2 + 0.010 A3, and A1 to A3 were the age (years), WBC (*10^9^/L), and DS (hours), respectively. ROC curve (Fig. 2) analysis of prediction model resulted in an AUC of 0.881 (95% CI: 0.845–0.915, *P* < 0.05). When the value of P was 0.62, the Youden index was the largest (0.65). Patients with the P of 0.62 or greater were considered to be more likely to have complicated appendicitis. The predictive values of prediction model in derivation sample were 82.8% sensitivity, 81.9% specificity, 84.8% positive predictive value (PPV) and 76.8% negative predictive value (NPV).

**Table 2:** Multivariate logistic regression analysis for complicated appendicitis (Derivation Sample: *n* = 382)

Variables	β	SE	0R	95% CI	*P* value
Age (years)	−1.559	0.208	0.210	0.140–0.316	< 0.001
WBC (*10^9^/L)	0.190	0.036	1.209	1.128–1.297	< 0.001
NEU (*109/L)	−0.101	0.080	0.904	0.773–1.058	0.209
LYMPH (*109/L)	0.099	0.080	1.104	0.944–1.292	0.214
PCT (ug/L)	0.076	0.043	1.079	0.993–1.173	0.072
CRP (mg/L)	0.003	0.003	1.003	0.997–1.009	0.325
DS (hours)	0.010	0.004	1.010	1.002–1.018	0.015
Body temperature (°C)	0.225	0.221	1.253	0.813–1.931	0.308
Migration of pain to RLQ	−0.382	0.542	0.682	0.236–1.975	0.481
Abdominal distention	−0.084	0.380	0.920	0.437–1.935	0.825
Rebound pain	1.263	0.495	3.537	0.840–9.333	0.091
Nausea/ vomiting	1.002	0.633	2.724	0.788–9.417	0.113
Diarrhea	0.828	0.658	2.288	0.630–8.313	0.209
Constant	2.997	0.976	20.026	–	0.002

β: regression coefficient; SE: standard error; OR: odds ratio; 95%CI: 95% confidence interval. *WBC* white blood cell count, *NEUT* neutrophil count, *LYMPH* lymphocyte count, *PCT* procalcitonin, *CRP* C-reactive protein, *DS* duration of symptoms, *LRQ* right lower quadrant

### Prediction model validation

Complete data for validation of the prediction model were available for 156 patients, 52.5% of whom had complicated appendicitis. In validation sample, the median age, WBC, and DS were significantly higher (age: 4.2 versus 3.5 [years], WBC, 15.6 versus 13.0 [*10^9^/L]; DS: 34 versus 17 [hours]; *P* < 0.05 for all) in patients with complicated appendicitis than that with simple appendicitis (Table 3). The optimal cutoff point was 0.62 for prediction model. The AUC for the prediction model in validation sample was 0.830 (95%CI: 0.762–0.885, *P* < 0.05) (Fig. 2). Our prediction model was shown to have a sensitivity of 77.8%, a specificity of 89.2%, a PPV of 88.7%, and an NPV of 77.6%. The diagnostic accuracy of the prediction model was 82.7%. The positive and negative likelihood ratios (LR) were 7.11 and 0.26, respectively.

**Table 3:** The clinical characteristics and scoring systems on the types of AA. (Validation Sample: *n* = 156)

Variables	Complicated appendicitis (*n* = 82)	Simple appendicitis (*n* = 74)	*P* value
**Clinical characteristics**			
Age (years)	3.5 (2.7,4.0)	4.2 (3.8,4.7)	< 0.001
WBC (*10^9^/L)	15.6 (14.1,18.4)	13.0 (9.7,15.5)	< 0.001
DS (hours)	34 (24,78)	17 (11,31)	< 0.001
**Scoring systems**			
PAS	7 (6,9)	5 (4,7)	< 0.001
ALVARADO score	8 (7,9)	6 (5,7)	< 0.001

*WBC* white blood cell count, *DS* duration of symptoms, *PAS* Pediatric Appendicitis Score

### Prediction model comparison

To compare the predictive value of ALVARADO score, PAS and prediction model, the ALVARADO score and PAS were calculated in validation sample. The median ALVARADO score and PAS were significantly higher (ALVARADO score: 8 versus 6, PAS: 7 versus 5, both *P* < 0.05) in patients with complicated appendicitis than that with simple appendicitis (Table 3).

**Fig. 3:**
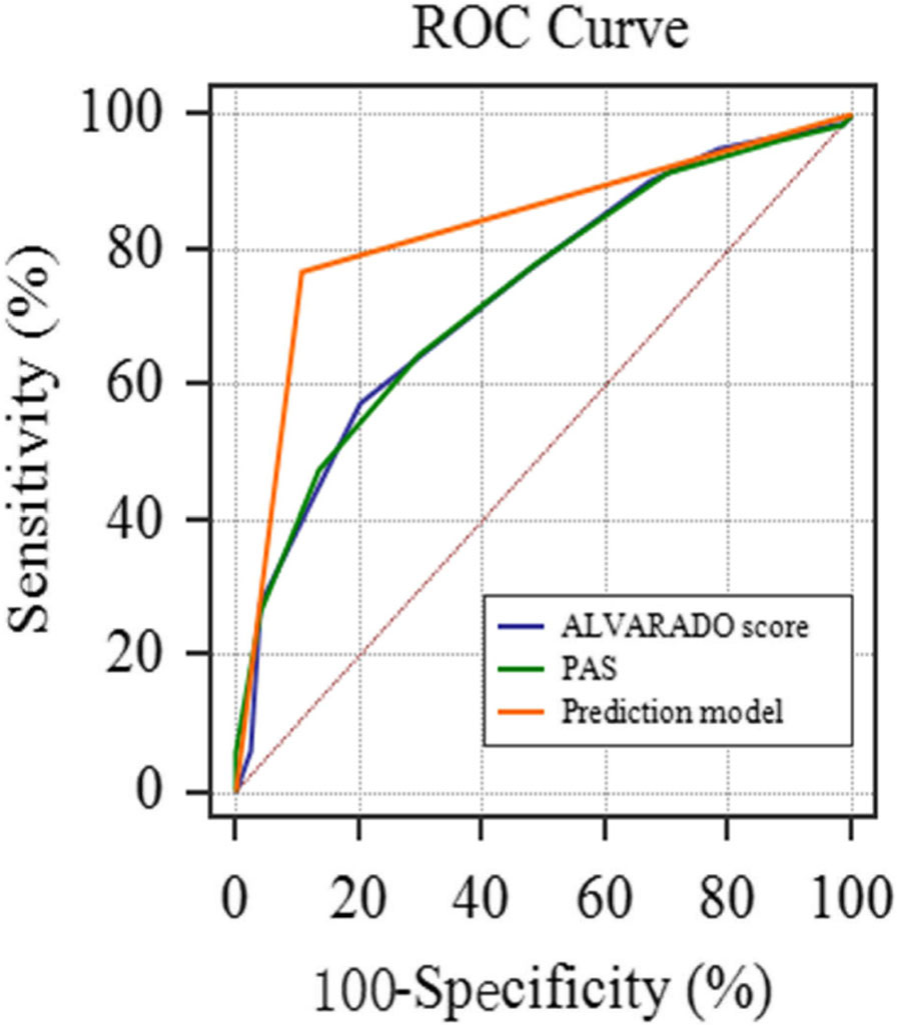
Comparison of the prediction model, ALVARADO score, and PAS in validation sample. The AUC for the prediction model was 0.830 (95% CI: 0.762–0.885), for ALVARADO score was 0.733 (95% CI: 0.657–0.801), for PAS was 0.735 (95% CI: 0.658–0.802)

In Fig. 3, The AUC for ALVARADO score was 0.733 (95% CI: 0.657–0.801) and that for PAS was 0.735 (95% CI: 0.658–0.802). The prediction model had an AUC greater than that for the ALVARADO score and PAS in validation sample (*P* < 0.05). No significant differences in AUC existed between the ALVARADO score and PAS (*P* > 0.05). When the score was 7 (optimal cutoff point), both ALVARADO score and PAS had the largest Youden index. In validation sample, patients with the score of 7 or greater were considered to be more likely to have complicated appendicitis. With the optimal cutoff point of 7, the discrimination values of ALVARADO score were 57.3% sensitivity, 79.7% specificity, 64.3% PPV and 67.2% NPV; the discrimination values of PAS were 64.6% sensitivity, 70.3% specificity, 70.7% PPV and 64.2% NPV (Table 4).

**Table 4:** Prediction model, ALVARADO score, and PAS performance at optimal cutoff point values (Validation Sample: *n* = 156)

	Optimal cutoff point	Sensitivity (%)	Specificity (%)	PPV (%)	NPV (%)	+LR	-LR
Prediction model	0.62	76.8	89.2	88.7	77.6	7.11	0.26
ALVARADO score	7	57.3	79.7	64.3	67.2	2.83	0.54
PAS	7	64.6	70.3	70.7	64.2	3.52	0.61

*PAS* Pediatric Appendicitis Score, *PPV* positive predictive value, *NPV* negative predictive value, *+LR* positive likelihood ratio, *−LR* negative likelihood ratio

## Discussion

In this retrospective study we found that age, WBC and DS on admission were independently associated with complicated appendicitis, and developed a prediction model based on these three independent predictors, aiming to make the discrimination of simple and complicated appendicitis in children younger than five years of age. Regarding prediction, the prediction model could identify children at high risk for complicated appendicitis, better than that of ALVARADO score and PAS. This model might be used to aid the differentiation of acute simple and complicated appendicitis for the optimal treatment strategy.

AA remains a clinical diagnosis with laboratory and radiological test as an auxiliary diagnostic method. Accurate differentiation between simple and complicated appendicitis is emerging as a potentially key issue as the historical standard of care, that is prompt appendectomy, is increasingly questioned in pediatric patients [7, 16]. Since AA has a rate of been complicated of approximately 40%, different methods for predicting complicated appendicitis have been tested with inconsistent results. Radiological tests and ultrasonography prove to have an approximately 20% of false negative complicated appendicitis. Both clinical and laboratory variables have been reported to be of value in diagnosing complicated appendicitis, but the results are equivocal in children younger than five years of age [7, 13, 17–19].

This study not only describe the independent risk factors for complicated appendicitis, but establish early identification of risk factors in order to predict complicated appendicitis. Thus, we included only those factors available in clinical database that were simple and easy to obtain. Based on the multivariate regression analysis results, we referred the Enter method to establish the prediction model. Even though DS were discussed in previous studies as well as in ours, we should notice that the factor is of subjective nature and its reproducibility is low [7]. Objective variables obtained from blood sample usually better reproducible and therefore of higher value. Among the variables included in our prediction model, DS is the only modifiable risk factor. Several studies have shown that longer DS of AA, the more likely it was to develop perforated [20–23]. Bickell et al. [20] reported the link between the duration of the symptoms and the probability of appendiceal perforation. They concluded that the chance of perforation is low in the first 36 h of the disease and increases by 5% every 12 h thereafter. We found a notable difference in the DS between the simple appendicitis and complicated appendicitis, which is why concluded that one of the reasons for high rates of complicated appendicitis in this age group could be a delayed visit to the doctor. Similar to our results, Bansal et al. [20] revealed notable differences in the DS between the groups of perforated and non-perforated appendicitis. However, we thought that due to the lack of intestinal barrier and underdeveloped omentum in children younger than five years of age, the DS had a more obvious effect on the appearance of gangrene and perforation in AA. This reminded us that shortening the DS may effectively avoid the probability of complicated appendicitis.

According to the requirements of the international transparent reporting of a multivariable prediction model for individual prognosis or diagnosis (TRIPOD) list and elaboration documents, the new prediction model needs to be verified by validation samples of the center or other centers in order to truly reflect the prediction performance of the model [24]. We collected clinical data of 156 cases for external verification, the discrimination was evaluated by calculating the AUC of ROC. When the cutoff point was 0.62, the AUC for the prediction model in validation sample was 0.830 (95% CI: 0.762–0.885). Our prediction model was shown to have a sensitivity of 77.8%, a specificity of 89.2%, a PPV of 88.7%, and an NPV of 77.6%. The diagnostic accuracy of our model in this cohort was high. In the 2 most commonly cited scores (ALVARADO score and PAS), the authors assign point values to patient history, physical examination, and laboratory findings [6]. In several studies, PAS and ALVARADO score could effectively diagnose complicated appendicitis [7, 25–27], but no research reported in patients younger than five years of age. We compare the predictive model with PAS and ALVARADO score for the differentiation of simple and complicated appendicitis. The prediction model had an AUC greater than that for the ALVARADO score or PAS in validation sample (*P* < 0.05). This may suggest that the ALVARADO score and PAS were not accurate enough to differentiate the type of AA in patients younger than five years of age. Therefore, the prediction model we made was a simple and efficient method that aids the differentiation of acute simple and complicated appendicitis.

Perforation in this age group often leads to diffuse peritonitis, and the most important thing in the management is to establish the accurate diagnosis and perform surgical treatment, assisted by broad-spectrum antimicrobial therapy [2, 21, 28]. Recently, several trials have focused on the non-operative treatment for AA [10, 29–31]. Studies suggested that different treatment strategies should be selected according to the type of AA: simple appendicitis should be the preferred antibiotic conservative treatment, while complicated appendicitis requires appendectomy in most cases [15, 32]. Children appendix is not a non-functional organ left in the body. The appendix is not only a “storage pool” for the gut microbiota to balance the steady state of the proinflammatory and anti-inflammatory activities of the intestine; and the high content of lymphoid tissue (mainly lymphocyte CD8+ T cells) in the appendix plays an important role in the immune function of the body [33, 34]. The age of 5 years and younger is an important period for children’s immune function to gradually mature and the balance of intestinal flora to establish. Conservative treatment for simple appendicitis can preserve the appendix, which not only helps maintain intestinal flora homeostasis and immune system development, but also reduces medical costs [16], [35]. Therefore, if the model shows that the patient has a high possibility of complicated appendicitis, an immediate appendectomy and broad-spectrum antimicrobial therapy may be necessary. And antibiotic conservative treatment priority strategies can be adopted to avoid unnecessary appendectomy for patients with simple appendicitis predicted by the model.

Furthermore, discrimination between simple and complicated appendicitis is important as it may guide appropriate intravenous fluid and antibiotic resuscitation prior to surgical intervention. The prediction model could guide preoperative (or postoperative) antibiotic selection and predict prognosis, referred the optimal cutoff point of 0.62. Children with simple appendicitis typically receive a single antibiotic preoperatively and may even not receive postoperative treatment and get discharge home relatively soon [13]. Conversely, children with a complicated appendicitis recognised on admission typically receive a combination of more antibiotics before appendectomy and continue antibiotic therapy postoperatively, and prolong the hospital duration of stay. Hence, identification of predictive indicators for the complicated appendicitis is essential.

It should be borne in mind that the present study was limited by its retrospective design and based on experiences within a single unit, further research with a larger prospective cohort study is necessary to validate the usefulness of the prediction model for predicting complicated appendicitis in children younger than five years of age. Furthermore, the definitions of simple and complicated appendicitis are based on the intraoperative findings and postoperative pathological results, and nonoperatively were excluded. It should be also worth noting that the normal values of WBC are affected by age, which was the inevitable limitation of this study.

## Conclusion

In conclusion, this study is the first to propose a clinical prediction model to predict complicated appendicitis in children younger than five years of age with AA, and the model showed fair predictive accuracy. Age, white blood cell count, and duration of symptoms could be used to predict complicated appendicitis in children younger than five years of age with acute appendicitis. However, further studies are required to improve the performance of the prediction model and increase sensitivity of complicated appendicitis.

## Data Availability

The datasets used and analysed during the current study are available from the corresponding author on reasonable request.

## Abbreviations

AA: Acute appendicitis
PAS: Pediatric Appendicitis Score
WBC: White blood cell count
DS: Duration of symptoms
AUC: Area under the curve
